# Bilateral ureteral obstruction revealing a benign prostatic hypertrophy: a case report and review of the literature

**DOI:** 10.1186/1752-1947-8-42

**Published:** 2014-02-11

**Authors:** Omar Riyach, Mustapha Ahsaini, Youssef Kharbach, Mohammed Bounoual, Mohammed Fadl Tazi, Jalal Eddine El Ammari, Soufiane Mellas, Mohammed El Jamal Fassi, Abdelhak Khallouk, Moulay Hassan Farih

**Affiliations:** 1 Department of Urology, University Hospital Center Hassan II, Fez, Morocco; 2 Faculty of Medicine and Pharmacy, Fez, BP: 1893 –Km 2.200 Route de Sidi Harazem, Fez, Morocco

**Keywords:** Bilateral hydronephrosis, Detrusor hypertrophy, Distal ureter obstruction, Prostatic hyperplasia

## Abstract

**Introduction:**

Prostatic hyperplasia is the most frequent tumor in men older than 50 years of age. Bilateral hydronephrosis secondary to benign prostatic hypertrophy is a rare condition most often due to vesicoureteral reflux. Herein we report a case of a patient with bilateral hydronephrosis with distal ureter obstruction caused by detrusor hypertrophy due to prostatic hyperplasia, our analysis of the clinical data and a review of the relevant published literature.

**Case presentation:**

We report a case of a 65-year-old Berber man with clinically significant storage, bladder-emptying symptoms and bilateral low back pain with renal biologic failure and bilateral ureterohydronephrosis, distal ureteral stenosis, detrusor hypertrophy and prostate hyperplasia without significant post-void residual urine volume visualized by abdominal sonography. The patient underwent bilateral JJ stent insertion with transurethral resection of the prostate. The patient was discharged 3 days after surgery without any obvious complications. At his 3-month follow-up examination, the JJ stent was removed and the patient had comfortable urination without renal failure.

**Conclusion:**

This is an extremely rare condition that has important diagnostic considerations because of the possibility of comorbid severe obstructive uropathy and chronic renal failure.

## Introduction

Benign prostatic hyperplasia (BPH) is the most common benign tumor in men. BPH is the growth of epithelial, muscular and/or fibrotic cells in the prostate [1] and is responsible for the occurrence of urinary symptoms in men older than 50 years of age. It is clinically manifested by a low obstructive uropathy syndrome, which includes storage and voiding symptoms, and is diagnosed on the basis of an adequate digital rectal examination (DRE), prostate and bladder ultrasound and prostate-specific antigen (PSA) level. In a complementary form, urinary flowmetry, cystoscopy and transrectal ultrasound can be tested, in addition to biopsy [2,3]. Intravenous urography (IVU) is a radiological tool for the assessment of BPH-related changes of the upper and lower urinary tracts (for example, hydronephrosis, trabeculation, diverticula and bladder stones) and other urological diseases (for example, nephro- and ureterolithiasis and upper urinary tract tumors). It has been abandoned in routine work-up of patients with BPH because of increased radiation risks, costs and little additional diagnostic benefit compared to ultrasonography [4,5]. However, it is still a valid tool to use to evaluate upper urinary tract modification associated to BPH, and an important diagnostic tool in cases of bilateral hydronephrosis without post-void residual (PVR), which is an extremely rare complication of this pathology that has been described in a few reported cases in the literature. So, herein we report a case of a patient with bilateral hydronephrosis with distal ureter obstruction caused by detrusor hypertrophy due to prostatic hyperplasia. We also analyzed the clinical data and reviewed the relevant published literature and present our findings.

## Case presentation

**Figure 1:**
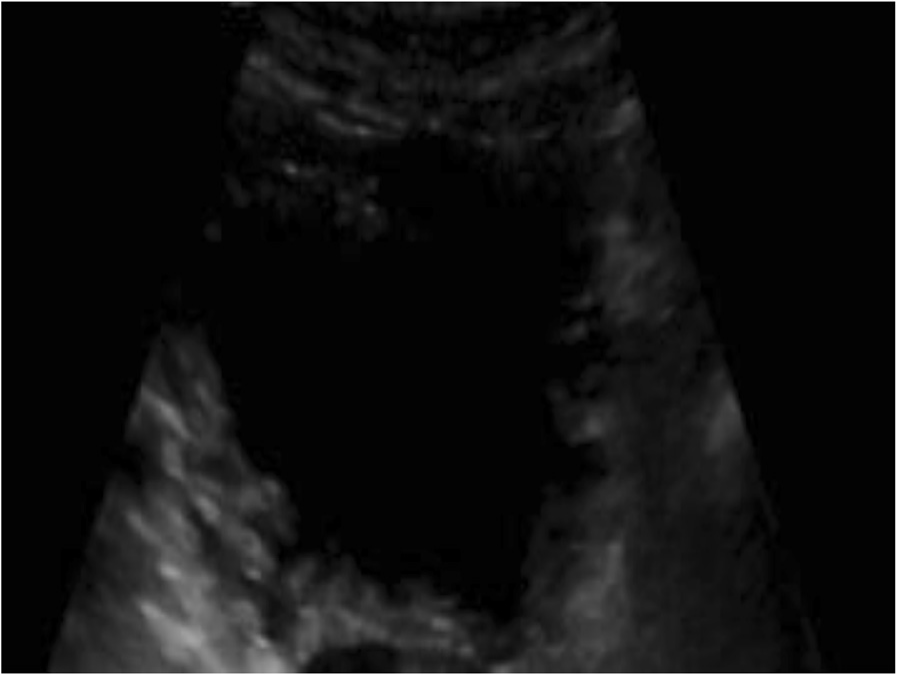
Bladder ultrasound showing trabeculation and bladder wall thickening.

**Figure 2:**
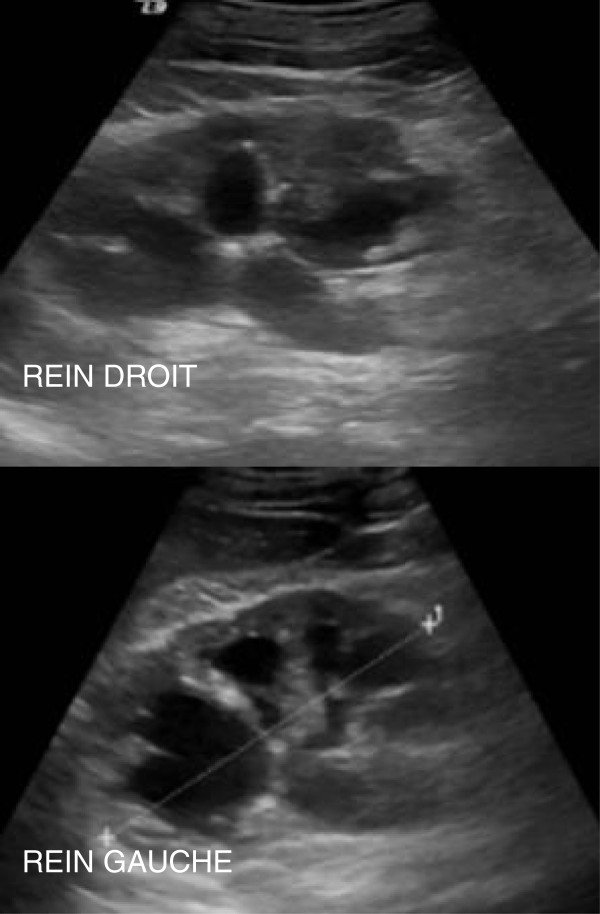
**Renal ultrasound images showing two bilateral ureterohydronephroses.** REIN DROIT: right kidney; REIN GAUCHE: left kidney.

**Figure 3:**
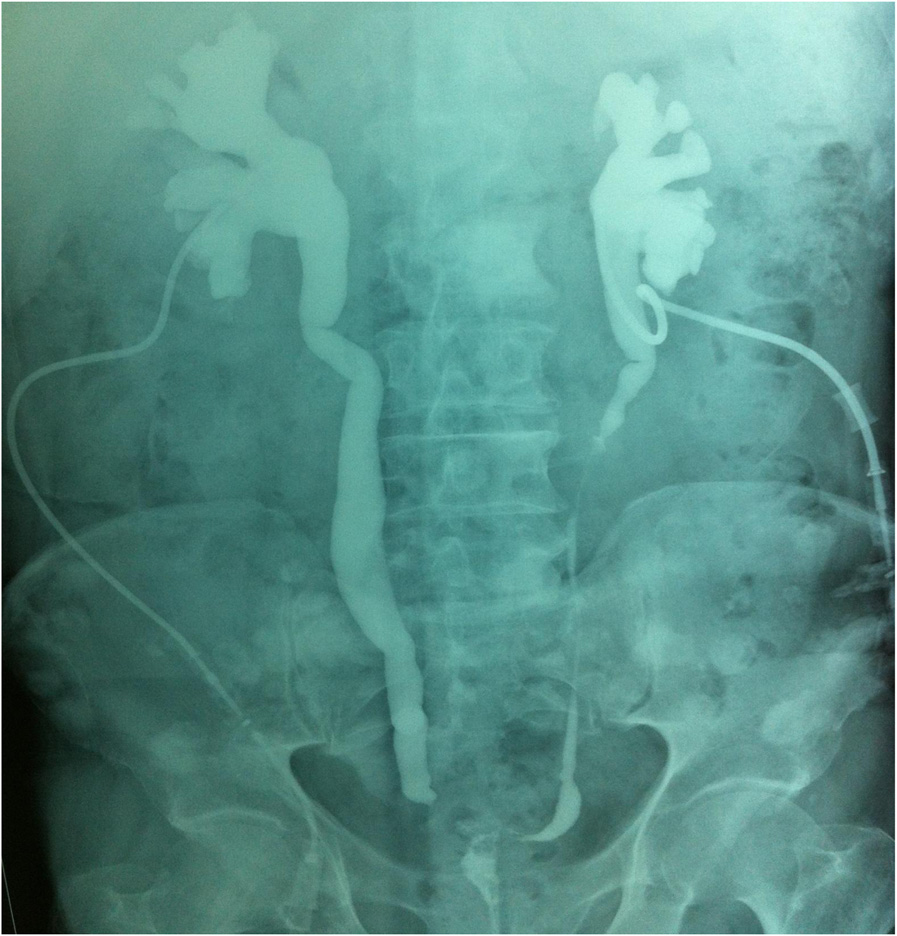
Drip infusion pyelogram showing important dilatation of the upper urinary tract with a partial defect of the distal ureter.

We report the case of a 65-year-old Berber man admitted to our urology department because of renal failure. He had a history of symptoms of bladder fullness and emptying with a 1-year evolution characterized by decreased force and caliber of the urine stream, intermittency, dysuria, frequent urge to urinate, urgency and terminal dribbling with nightly urination (three to five times). The evolution of his symptoms included acute urinary retention 1 month prior to urological testing. During the patient’s medical history interview, he referred to chronic lumbar pain but no weight loss. During the physical examination, his full bladder was found not to be palpated, his genitals were adequate for age and sex and a DRE revealed a normotonic sphincter. His prostate was enlarged and had an adenomatous surface. The routine laboratory findings revealed biologic renal failure with the following laboratory panel values: urea 43.5mg/dl, creatinine 0.8mg/dl, sodium 144mEq/L, potassium 4.5mEq/L, chloride 118mEq/L and free PSA 4.12ng/ml. An abdominal ultrasound confirmed prostatic enlargement, estimated at 45ml with regular edges, good definition of homogeneous echogenicity and regular bladder wall thickness hypertrophy (Figure 1). Another image showed bilateral ureterohydronephrosis (Figure 2), and PVR urine volume was found to be 35ml. Uroflowmetry demonstrated maximum urinary flow rate and average urinary flow rate to be 13ml/s. Our investigations were completed with drip infusion pyelography, which showed significant dilatation of the upper urinary tract and a partial defect of the distal ureter (Figure 3).

The patient underwent bilateral JJ stent insertion with transurethral resection of the prostate. The Foley catheter was removed after 2 days, after which the patient was able to void without difficulty. The patient was discharged 3 days after surgery without any obvious complications. A pathologic examination revealed benign prostatic gland hyperplasia. At the patient’s 3-month follow-up examination, his JJ stent was removed. He had comfortable urination without renal failure.

## Discussion

BPH is a common problem experienced by aging men that can lead to serious outcomes, including acute urinary retention and bladder stone formation. Its prevalence is directly proportional to increase in age. At 80 years of age, its prevalence is 95% [3]. These prostatic changes begin at 40 years of age, and prostate volume increases by around 0.6ml/yr and is associated with a reduction of mean urinary flow at the rate of 0.2ml/s [6]. This is not necessarily definitive, because prostate growth and the severity of its symptoms are erratic in the patient population. Known as prostatism, this constellation of clinical symptoms is characterized by the presence of irritable and obstructive lower urinary tract symptoms, which are evaluated in a general manner on the basis of the International Prostate Symptom Score. Chronic or acute obstructive renal failure is a serious condition associated with premature mortality, decreased quality of life and increased health-care expenditures. Both diseases are extremely common among aging men, leading some to suggest that it is a natural concomitant of aging. Nonetheless, quite recently, evidence of an association between BPH and chronic kidney disease (CKD) has arisen in two different studies. In a study by Yamasaki *et al*. [7], the PVR of patients with CKD was significantly greater than that of patients without CKD, and the presence of PVR urine was independently associated with CKD, indicating a close association between CKD and residual urine. In that study, PVR was used as a surrogate measure of bladder outlet obstruction and thus of urodynamically relevant BPH [7]. Yamasaki *et al*. reported a higher prevalence (31.8%) of CKD among BPH patients than those without BPH. Although the prevalence of CKD can be considered relatively low among men with BPH, the possibility of CKD should be considered in those who have a low maximum flow rate and obstructive urinary symptoms. Chronic urinary retention is thought to be the dominant mechanism by which BPH can cause chronic renal failure, Rule *et al*. defined chronic urinary retention (CUR) as PVR urine levels higher than 100ml and reported that CUR was significantly associated in CKD in community-dwelling men [8-10].

For years, it has been well-described that large urinary volume (greater than 300ml) affects renal function in patients with advanced BPH. To the best of our knowledge, the case of our patient involved the largest urinary volume reported in the literature. In our patient, the ureterovesical junction obstruction caused by detrusor hypertrophy seems to have been the principal contributing factor to renal failure in BPH. Upper tract dilation occurs as a consequence of a continuum of bladder outlet obstruction and remodeling (detrusor hypertrophy and scarring), leading to anatomical ureterovesical junction obstruction. Upper urinary tract dilation or hydronephrosis is consistent with chronic renal failure due to obstructive uropathy. In men with BPH and increased serum creatinine levels, hydronephrosis is common (occurring in one-third of patients), with a prevalence of 90% in men with BPH who are hospitalized for uremic symptoms. In ultrasound evaluation of patients with bilateral hydroureteronephrosis, it is common to observe compressing and thinning of the renal cortex with obvious impact on renal function. Imaging studies are an excellent diagnostic tool. These tools include ultrasonography and excretory urography. Because IVU has not routinely been conducted in patients with creatinine levels (180mmol/L), patients without impaired renal function still represent the best examples for showing this relationship by revealing bilateral upper urinary tract dilation with regular ureterovesical junction obstruction. Transurethral resection of the prostate is still the gold standard treatment of benign prostatic hypertrophy, even in cases with associated hydronephrosis and ureterovesical junction obstruction caused by detrusor hypertrophy [11]. The surgical treatment options for BPH have dramatically changed with the development of minimally invasive therapies over the past two decades. They include holmium laser enucleation of the prostate, transurethral electrovaporization of the prostate, transurethral microwave thermotherapy and other modalities [11]. However, these techniques are also performed in patients with slightly to moderately enlarged prostates. Rocco *et al*. stated that 100ml is regarded as the prostate weight limit for those minimally invasive procedures [12]. European Association of Urology guidelines state that open prostatectomy is the treatment of choice for large prostate glands more than 80ml to 100ml in size [13]. If ureterohydronephrosis and azotemia persist despite bladder unblocking, ureterovesical junction obstruction should be considered, and bilateral percutaneous nephrostomy or bilateral ureteric stents are advisable for temporary drainage, as we have reported in our present case. We think that our case is interesting and rare, particularly with regard to the patient’s lumbar pain due to bilateral ureterohydronephrosis as the first and principal symptom of benign prostatic hypertrophy.

## Conclusion

The physiological causes that lead to the ureterovesical junction obstruction in BPH are still incompletely known. It is a rare disease; nevertheless, its diagnosis and early treatment are important because of its association with severe obstructive uropathy and chronic renal insufficiency.

## Consent

Written informed consent was obtained from the patient for publication of this case report and any accompanying images. A copy of the written consent is available for review by the Editor-in-Chief of this journal.

## Abbreviations

BPH: Benign prostatic hyperplasia; CKD: Chronic kidney disease; CUR: Chronic urinary retention; DRE: Digital rectal examination; IVU: Intravenous urography; PVR: Post-void residual.

## Competing interests

The authors declare that they have no competing interests.

## Authors’ contributions

OR and MA are the principal authors and made major contributions to the writing of the manuscript. YK, MB, MFT, JE, SM, MJE, AK and MHF analyzed and interpreted the patient data and reviewed the literature. All authors read and approved the final manuscript.
